# Notoginsenoside R1 Alleviates Oxygen–Glucose Deprivation/Reoxygenation Injury by Suppressing Endoplasmic Reticulum Calcium Release via PLC

**DOI:** 10.1038/s41598-017-16373-7

**Published:** 2017-11-24

**Authors:** Yan Wang, Liu Tu, Yingbo Li, Di Chen, Zhao Liu, Xuelian Hu, Shali Wang

**Affiliations:** 1 Cerebrovascular Diseases Laboratory, Institute of Neuroscience, Chongqing Medical University, Chongqing, 400016 China; 2 Department of Neurology, Chongqing General Hospital, Chongqing, 400014 China; 3 Department of Pharmacy, Second Affiliated Hospital, Third Military Medical University, Chongqing, 400038 China

## Abstract

As documented in our previous study, notoginsenoside R1 (NGR1) can inhibit neuron apoptosis and the expression of endoplasmic reticulum (ER) stress-associated pro-apoptotic proteins in hypoxic–ischemic encephalopathy. Recent evidence indicates that the Phospholipase C (PLC)/inositol 1,4,5-trisphosphate receptor (IP3R) is important for the regulation of Ca^2+^ release in the ER. Ca^2+^ imbalance can stimulate ER stress, CAMKII, and cell apoptosis. The purpose of this study was to further investigate the neuroprotective effect of NGR1 and elucidate how NGR1 regulates ER stress and cell apoptosis in the oxygen–glucose deprivation/reoxygenation (OGD/R) model. Cells were exposed to NGR1 or the PLC activator m-3M3FBS. Then, IP3R- and IP3-induced Ca^2+^ release (IICR) and activation of the ER stress and CaMKII signal pathway were measured. The results showed that NGR1 inhibited IICR and strengthened the binding of GRP78 with PERK and IRE1. NGR1 also alleviated the activation of the CaMKII pathway. Pretreatment with m-3M3FBS attenuated the neuroprotective effect of NGR1; IICR was activated, activation of the ER stress and CaMKII pathway was increased, and more cells were injured. These results indicate that NGR1 may suppress activation of the PLC/IP3R pathway, subsequently inhibiting ER Ca^2+^ release, ER stress, and CaMKII and resulting in suppressed cell apoptosis.

## Introduction

Hypoxic–ischemic encephalopathy (HIE) is a severe brain disease with high morbidity and mortality worldwide^1^. Typically, HIE causes irreversible necrosis, cell apoptosis, and neuron death, which can lead to permanent neurological morbidity^2^. Our previous study revealed that notoginsenoside R1 (NGR1) protects the brain from hypoxic–ischemic injury through the inhibition of endoplasmic reticulum (ER) stress pathways^3^. The present experiments were designed to determine the mechanism for NGR1 action on ER stress and other apoptosis pathways.

ER stress that initiates apoptotic signaling has been implicated in hypoxia–ischemia^4^. Glucose-regulated protein 78 (GRP78) is one of the initial components in the signaling cascade that results in ER stress^5^. Studies have shown that GRP78 binds with double-stranded RNA-activated protein kinase-like endoplasmic reticulum kinase (PERK) and inositol-requiring enzyme 1 (IRE1) to impede the accretion of unfolded proteins in cells or crosses the karyolemma to raise the transcription of effector proteins for ER stress^6^. Under persistent stress, GRP78 dissociates from IRE1/PERK, which lead to the activation of ER stress^7^. The activation of PERK and IRE1 leads to cell apoptosis^8^. Phospho-PERK can trigger the expression of CCAAT/enhancer-binding protein homologous protein (CHOP), and phospho-IRE1 can inhibit B-cell lymphoma-2 (BCL-2)^9^.

It is well known that the loss of Ca^2+^ homeostasis plays an important role in ischemia-induced neuronal damage^10,11^ upon oxygen–glucose deprivation/reoxygenation (OGD/R), a well-established *in vitro* model of ischemia^12^. In this model, substantial Ca^2+^ is released from the ER, resulting in an increased Ca^2+^ concentration in the cytosol^13^. Severe Ca^2+^ depletion of the ER leads to GRP78 dissociation from PERK and IRE1, which causes the subsequent activation of ER stress and cell apoptosis^14,15^. We hypothesize that NGR1 may protect neurons from OGD/R injury via inhibition of the dysregulation of ER Ca^2+^ following ischemia.

Phospholipase C (PLC) is an enzyme located on the nuclear envelope. Receptors for extracellular stimuli promote the activation of PLC, which results in the hydrolysis of phosphatidylinositol 4,5-bisphosphate (PIP2) to inositol 1,4,5-trisphosphate (IP3) and diacylglycerol (DAG)^16^. The IP3 receptor (IP3R) is a ubiquitously expressed Ca^2+^-release channel on the ER. There are 3 subtypes of IP3R (IP3R1, IP3R2, and IP3R3) in mammals, and each isoform of IP3R has its own characteristic expression pattern *in vivo*. Among the 3 subtypes, IP3R1 is a brain-dominant subtype^17^. Under stress, Ca^2+^ mobilization can be induced by the bonding of IP3 to IP3R^18^; further, Ca^2+^ release through the IP3R leads to the depletion of the ER’s Ca^2+^ and an increased Ca^2+^ concentration in the cytoplasm. Disturbance of Ca^2+^ homeostasis in the ER causes ER stress and activates cell injury^19^. Ma *et al*. found that PLC activity can increase Ca^2+^, thereby activating the apoptotic signal pathway^20^. Furthermore, severe increases in cytosolic Ca^2+^ concentration could stimulate Ca^2+^/calmodulin-dependent protein kinase II (CaMKII)^21^. CaMKII, an important member of the calcium/calmodulin-activated protein kinase family, plays a vital role in the regulation of both neuronal death and neuronal survival^22^. When Ca^2+^ combines with CaMKII, CaMKII is activated. Several studies have demonstrated that the phosphorylation of CaMKII plays an important role in cell death following an acute excitotoxic insult^23,24^. The phosphorylation of CaMKII also activates the c-Jun N-terminal kinase (JNK) and p38^25^. JNK and p38 are activated in response to a variety of stress signals and are implicated in death receptor-initiated extrinsic as well as intrinsic mitochondrial apoptotic pathways^26^.

The above findings suggest that inhibiting the activation PLC/IP3R may be effective for regulating the ER Ca^2+^–ER stress and ER Ca^2+^–CaMKII signal pathway and cell apoptosis. Our previous study demonstrated that NGR1 exerts neuroprotective effects by suppressing ER stress. The present study sought to build on this by further elucidating the neuroprotective effect of NGR1 and determining whether the ER Ca^2+^–ER stress and ER Ca^2+^–CaMKII signaling pathway is involved in this neuroprotective effect. In this study, multiple approaches were employed to explore the neuroprotective effects of NGR1 against OGD/R injuries in primary cortical neuron cultures and identify the underlying mechanisms. To the best of our knowledge, this study was the first to show that the neuroprotective effects of NGR1 against OGD/R may suppress the activation of the PLC pathway to regulate the ER stress and CaMKII signal pathway.

## Results

### NGR1 inhibited the activation of PLC/IP3R1 after OGD/R

**Figure 1:**
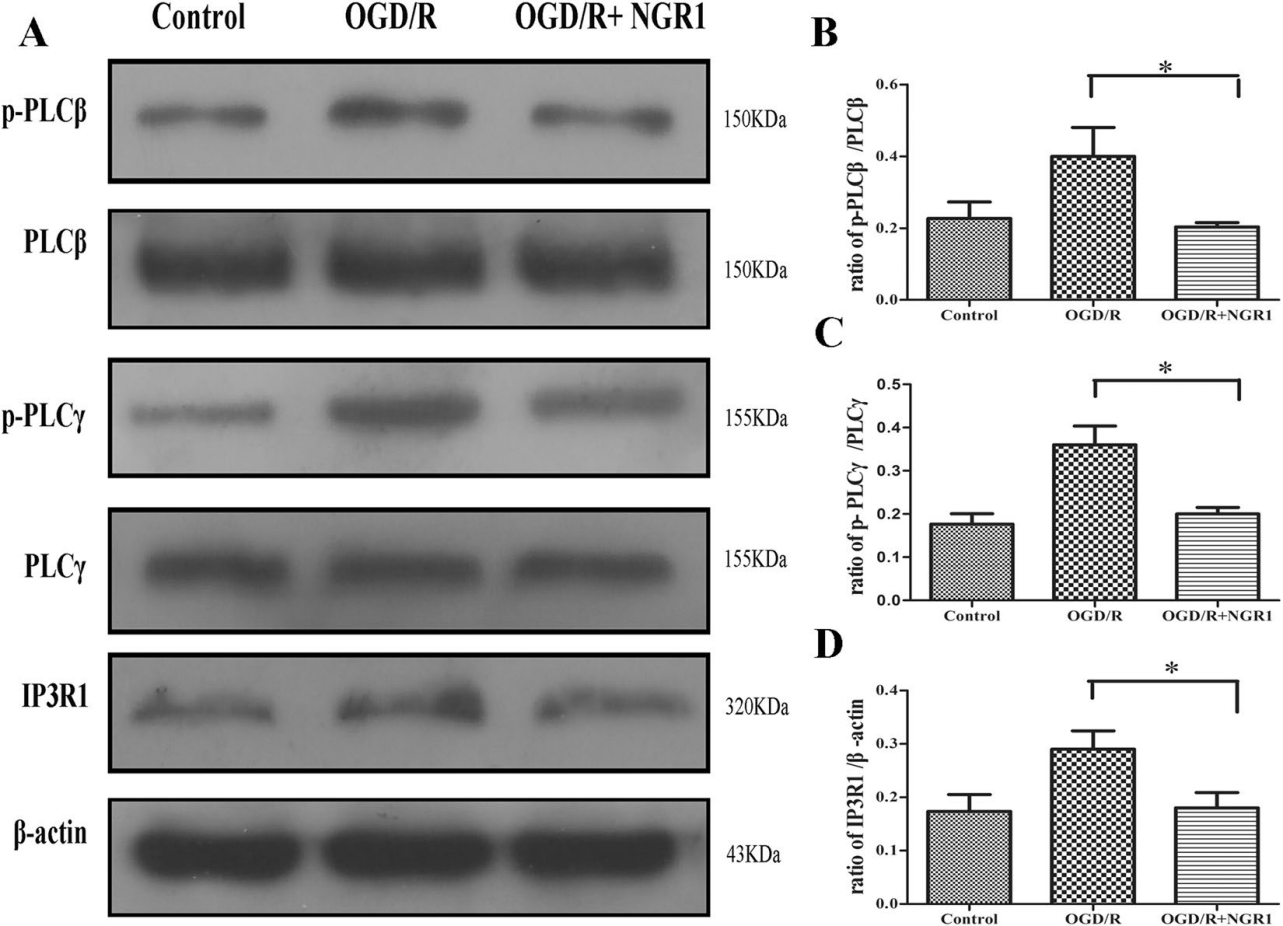
NGR1 attenuated OGD/R-induced stimulation of PLC and IP3R1. (**A**) Western blotting analysis of p-PLCβ, PLCβ, p-PLCγ, PLCγ, and IP3R1. (**B**–**D**) The relative protein expressions of p-PLCβ to PLCβ, p-PLCγ to PLCγ, and IP3R1 to β-actin are illustrated in the bar graphs. In the OGD/R case, the expressions of p-PLCβ, p-PLCγ, and IP3R1 were increased. NGR1 attenuated the stimulation of p-PLCβ, p-PLCγ, and IP3R1. Original image of the cropped blots shown in Supplementary Figure S2. *p < 0.05 compared with the other groups, n = 5, data presented as mean ± *SD*.

P-PLCβ, PLCβ, p-PLCγ, PLCγ, and IP3R1 expression was detected by Western blotting upon the stimulation of OGD/R or OGD/R + NGR1. OGD/R treatment increased the expression of p-PLCβ and p-PLCγ compared to the control group, but NGR1 decreased the phosphorylation of PLCβ and PLCγ (Fig. 1). NGR1 led to lower expression of IP3R1 compared with the OGD/R group.

### NGR1 strengthened the cooperation between GRP78 and PERK/IRE1 by suppressing PLC

**Figure 2:**
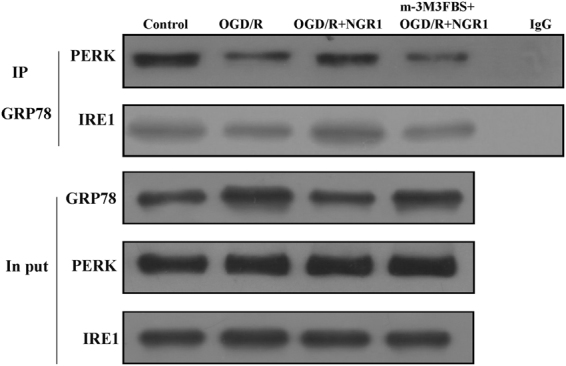
NGR1 strengthened the cooperation between GRP78 and PERK/IRE1. Coimmunoprecipitation was used to measure the bonds between GRP78 and PERK/IRE1. OGD/R weakened the bonds between GRP78 and PERK/IRE1. NGR1 strengthened the bonds between GRP78 and PERK/IRE1, while the function of NGR1 was blocked by m-3M3FBS, and PERK/IRE1 binding with GRP78 was reduced in the OGD/R + NGR1 + m-3M3FBS group. Original image of the cropped blots shown in Supplementary Figure S3.

To detect whether NGR1 could  strengthen the cooperation between GRP78 and PERK/IRE1 via PLC, cells were exposed to m-3M3FBS 1 h before OGD/R. As shown in Fig. 2, OGD/R weakened the binding of GRP78 and PERK/IRE1, and NGR1 treatment enhanced the binding of GRP78 and PERK/IRE1; however, in the OGD/R + NGR1 + m-3M3FBS group, the binding of GRP78 and PERK/IRE1 was weaker than that in the OGD/R + NGR1group. These results show that NGR1 can promote GRP78 binding with PERK and IRE1 by suppressing PLC activation.

### NGR1 inhibited endoplasmic reticulum Ca^2+^ release by suppressing PLC

To further explore the relationship among NGR1, PLC, and ER Ca^2+^, m-3M3FBS (20 μmol/L) was used to stimulate the activation of PLC.

**Figure 3:**
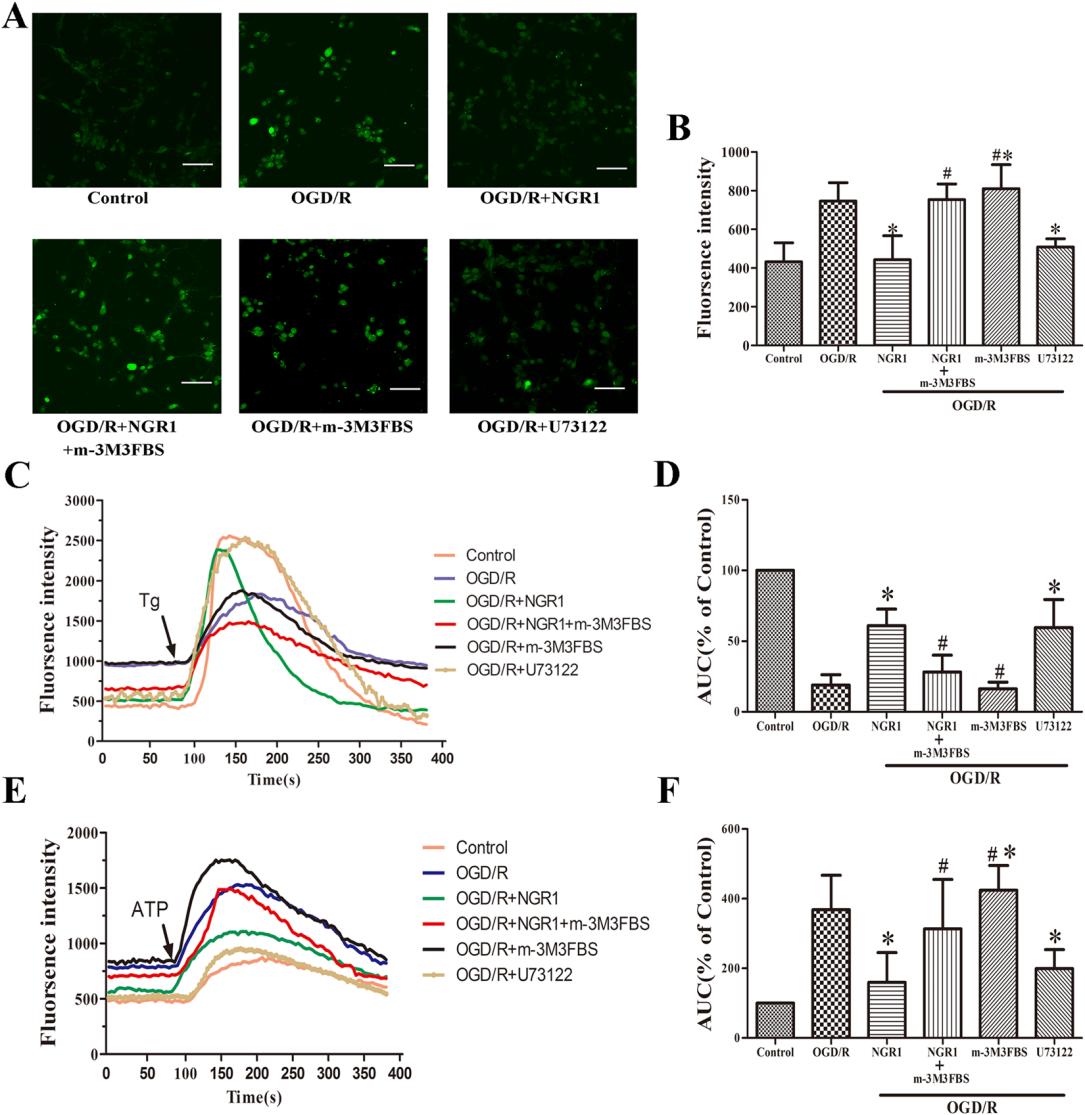
NGR1 attenuated OGD/R-induced endoplasmic reticulum calcium depletion by suppressing PLC. (**A**,**B**) The fluorescence intensity of cells was used to represent the Ca^2+^ concentration in the cytoplasm. There was more Ca^2+^ in the cytoplasm of the OGD/R group than in that of the control group, but the OGD/R + NGR1 and OGD/R + U73122 groups had lower Ca^2+^ concentrations than the OGD/R group, with a higher concentration of Ca^2+^ in the OGD/R + NGR1 + m-3M3FBS group. M-3M3FBS alone induced higher cytoplasm Ca^2+^ compared with the OGD/R group. (**C**,**D**) Thapsigargin (0.2 μM) was used to trigger the release of Ca^2+^ from the endoplasmic reticulum. Less Ca^2+^ was stored in the endoplasmic reticulum of the OGD/R group than in that of the control group, but in the OGD/R + NGR1 and OGD/R + U73122 groups, more Ca^2+^ was stored in the endoplasmic reticulum. In the OGD/R + NGR1 + m-3M3FBS group, Ca^2+^ was depleted and less Ca^2+^ release was triggered by Thapsigargin. In the OGD/R + m-3M3FBS group, less Ca^2+^ was stored in the endoplasmic reticulum. (**E**,**F**) ATP (2 μM) could trigger IP3-induced Ca^2+^ release (IICR), and ATP could induce more Ca^2+^ release from the endoplasmic reticulum in the OGD/R group than in the OGD/R + NGR1 and OGD/R + U73122 groups. In the OGD/R + NGR1 + m-3M3FBS group, more Ca^2+^ was released from the IP3R1 compared with the OGD/R + NGR1 group. M-3M3FBS alone induced more Ca^2+^ release than in the OGD/R and OGD/R + NGR1 groups. Scale bar = 50 μm, *p < 0.05 compared with the OGD/R groups, ^#^ *p* < 0.05 compared with the OGD/R + NGR1 groups, n = 5, data presented as mean ± *SD*.

The concentration of Ca^2+^ in the cytoplasm was detected, and the results showed that NGR1 could independently inhibit the increase in cytoplasm Ca^2+^ induced by OGD/R treatment, but the OGD/R + NGR1 + m-3M3FBS group had more Ca^2+^ in the cytoplasm than the OGD/R + NGR1group. The OGD/R + m-3M3FBS group had more cytoplasm Ca^2+^ than the OGD/R group, indicating that m-3M3FBS treatment could independently aggravate calcium overload. NGR1 and U73122 (an inhibitor of PLC) decreased the concentration of cytoplasm Ca^2+^ (Fig. 3A,B).

Endoplasmic reticulum lumenal Ca^2+^ was assessed by recording thapsigargin (Tg)-releasable calcium. Tg (0.2 μmol/L), an ER Ca^2+^-ATPase inhibitor, was applied to neurons to measure releasable Ca^2+^ from the ER stores in the absence of extracellular Ca^2+^. Tg triggered a transient increase of Ca^2+^ in the cytoplasm that was resolved in 1–3 min (Fig. 3C). The integrated area under the Ca^2+^ curve indicated the content of ER Ca^2+^ stores. The results showed that ER Ca^2+^ stores were lower after OGD/R but there were higher Ca^2+^ in the OGD/R + NGR1 group. However, the ER Ca^2+^ were lower in the OGD/R + NGR1 + m-3M3FBS group than in the OGD/R + NGR1group (Fig. 3C,D). Moreover, m-3M3FBS alone could promote Ca^2+^ release from the ER; in the OGD/R + m-3M3FBS group, the Ca^2+^ in the ER was smaller than that in the OGD/R group. U73122 showed the opposite effect—there was more Ca^2+^ stored in the ER in the OGD/R + U73122 group than in the OGD/R group.

To further examine the kinetic changes in ER Ca^2+^, we measured IP3-induced Ca^2+^ release (IICR) using ATP (2 μmol/L) to trigger the Ca^2+^ release in the absence of extracellular Ca^2+^ (Fig. 3E,F). In the OGD/R group, the Ca^2+^ release from the ER was higher than that in the control group, indicating that IICR was activated by OGD/R, but in the OGD/R + NGR1 group, the Ca^2+^ release from the ER was lower than that in the OGD/R group, showing that NGR1 inhibited the IICR. Importantly, in the OGD/R + NGR1 + m-3M3FBS group, IICR was easily triggered by ATP (Fig. 3E,F). Additionally, under OGD/R, NGR1 inhibited Ca^2+^ release from the ER through IP3R1, but when PLC was activated by m-3M3FBS, the effect of NGR1 was suppressed.

### NGR1 regulated the ER stress and CaMKII pathway via PLC

**Figure 4:**
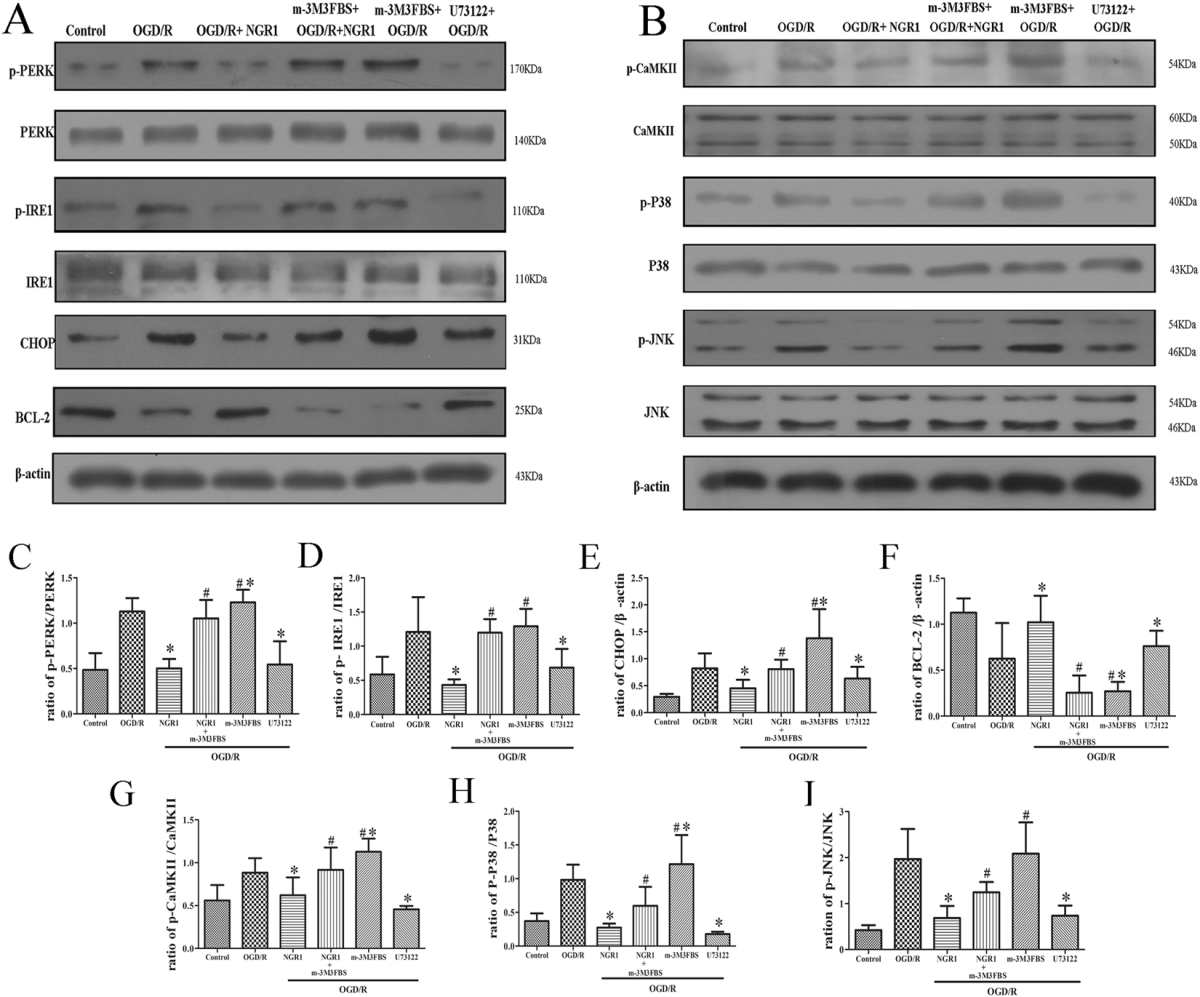
NGR1 inhibited OGD/R-induced stimulation of the ER stress and CaMKII pathway via PLC. (**A**,**B**) Western blotting analysis of ER stress and CaMKII pathway-related proteins. (**C**–**I**) When cells were exposed to NGR1 or U73122, there was less expression of p-PERK, p-IRE1, CHOP, p-CaMKII, p-P38, and p-JNK and more expression of BCL-2, but in the OGD/R + NGR1 + m-3M3FBS group, higher expression of p-PERK, p-IRE1, CHOP, p-CaMKII, p-P38, and p-JNK and lower expression of BCL-2 were detected. M-3M3FBS treatment could induce higher activation of ER stress and CaMKII than that in the OGD/R group. Original image of the cropped blots shown in Supplementary Figure S4. *p < 0.05 compared with the OGD/R groups, ^#^ *p* < 0.05 compared with the OGD/R + NGR1 groups, n = 5, data presented as mean ± *SD*.

P-PERK, p-IRE1, CHOP, p-CaMKII, p-p38, and p-JNK expression was higher and BCL-2 expression was lower in the OGD/R group than in the control group, but NGR1 inhibited the activation of p-PERK, p-IRE1, CHOP, p-CaMKII, p-p38, and p-JNK and promoted the expression of BCL-2. In the OGD/R + U73122 group, U73122 also significantly inhibited the expression of ER stress-related proteins and CaMKII pathway-related proteins. To detect whether NGR1 could suppress the activation of the ER stress and CaMKII pathway through PLC, cells were exposed to m-3M3FBS 1 h before OGD/R. As shown in Fig. 4A, p-PERK, p-IRE1, and CHOP expression was higher and BCL-2 expression was lower in the OGD/R + NGR1 + m-3M3FBS group than in the OGD/R + NGR1 group. These results suggested that NGR1 can inhibit the activation of ER stress via PLC. Under the same treatment, we measured the expressions of p-CaMKII, p-p38, and p-JNK and found higher p-CaMKII, p-p38, and p-JNK expression in the OGD/R + NGR1 + m-3M3FBS group than in the OGD/R + NGR1 group (Fig. 4B). These results show that NGR1 can also inhibit the activation of the CaMKII pathway via PLC. Moreover, m-3M3FBS treatment alone under OGD/R could aggravate the activation of the ER stress and CaMKII pathway. At the same time, one group of cells were exposed to U73122 (an inhibitor of PLC), 1 h before OGD/R, the results show that U73122 could also inhibit the activation of ER stress and CaMKII as NGR1.

### NGR1 showed neuroprotective effects via PLC

**Figure 5:**
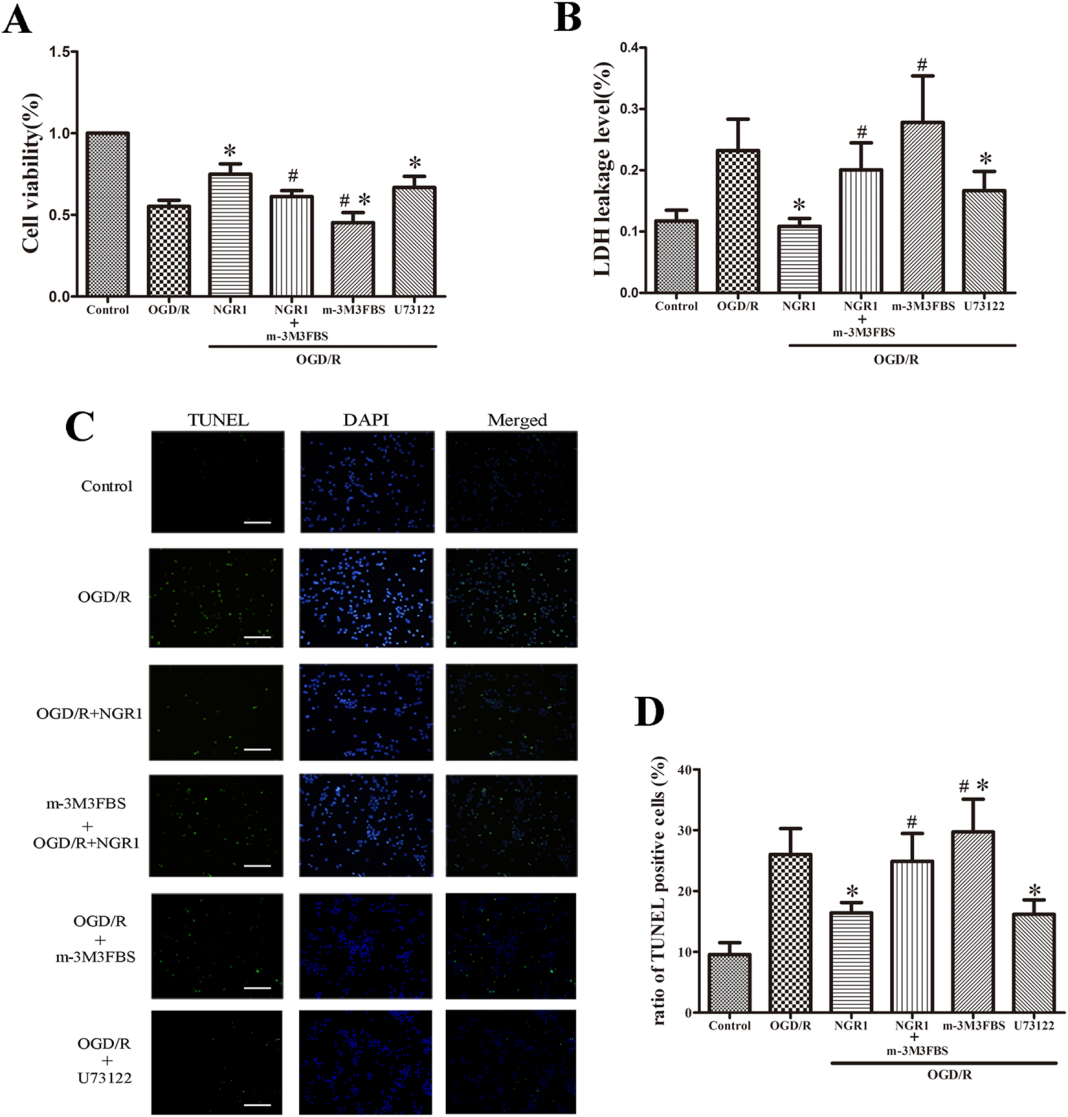
NGR1 reduced cell apoptosis via PLC. (**A**) The MTT assay showed that m-3M3FBS pretreatment abolished the neuroprotective effects of NGR1, and there was lower cell viability with m-3M3FBS treatment in the OGD/R + NGR1 + m-3M3FBS group than in the OGD/R + NGR1 group; m-3M3FBS treatment independently aggravated cell injury. (**B**) LDH leakage was higher in cells exposed to m-3M3FBS than in the OGD/R + NGR1 group; m-3M3FBS treatment independently aggravated LDH leakage. (**C** and **D**) TUNEL-positive nuclei are indicated by green fluorescent staining, and total nuclei are indicated by blue DAPI staining. M-3M3FBS significantly blocked the neuroprotective effects of NGR1. There were more TUNEL-positive cells in the OGD/R + NGR1 + m-3M3FBS group than in the OGD/R + NGR1 group. m-3M3FBS treatment alone led to more TUNEL-positive cells, and U73122 could inhibit cell apoptosis after OGD/R. Scale bar = 100 μm, *p < 0.05 compared with the OGD/R groups, ^#^ *p* < 0.05 compared with the OGD/R + NGR1 groups, n = 5, data presented as mean ± *SD*.

M-3M3FBS was used to activate PLC, and cells were measured using an MTT assay, LDH release, and TUNEL staining at 24 h after OGD/R (Fig. 5). The results showed that NGR1 (10 μmol/L) and U73122 (2 μmol/L) decreased cell apoptosis and LDH leakage, and the effect of NGR1 was blocked by m-3M3FBS. In the OGD/R + NGR1 group, the cell viability was higher and the LDH leakage and number of TUNEL-positive cells were lower than in the OGD/R group, but m-3M3FBS treatment inhibited the effect of NGR1; compared with the OGD/R + NGR1 group, the cell viability was lower and the LDH leakage and number of TUNEL-positive cells were higher in the OGD/R + NGR1 + m-3M3FBS group. M-3M3FBS treatment alone aggravated OGD/R-induced cell injury; in the OGD/R + m-3M3FBS group, the cell viability was lower and the LDH leakage and number of TUNEL-positive cells were higher in than in the OGD/R group. These results show that NGR1 exerts its neuroprotective effects via PLC.

## Discussion

Hypoxic–ischemic injury leads to the expansive neuron death and plays a key role in the development of neuron apoptosis^27^. NGR1 is isolated from *Panax notoginseng*, which possesses many therapeutic properties, including antiapoptotic, anti-inflammatory, and neuroprotective. Studies have found that NGR1 can regulate the inflammatory reaction through the NF-κB pathway^28^, reduce myocardial ischemia injury^29^, and inhibit the accumulation of β-amyloid proteins in mice with Alzheimer’s disease^30^. Studies have demonstrated that ER stress occurs in ischemia–hypoxia injury, inducing cell apoptosis, and the regulation of ER stress can decrease the vulnerability of neurons to HIE^31,32^. Our previous study found that NGR1 attenuates OGD-induced neuron injury by inhibiting the activation of ER stress^3^; similarly, Yu *et al*. found that NGR1 inhibits the hypoxia–reoxygenation-induced ER stress in H9c2 cardiomyocytes^33^. In the present study, we aimed to elucidate the action of NGR1 on ER stress.

We used primary cortical neurons to build an OGD/R model to mimic HIE, and we exposed cells to NGR1, m-3M3FBS (a PLC activator), and U73122 (a PLC inhibitor). Western blotting, coimmunoprecipitation, calcium imaging, and TUNEL staining were used to estimate the kinetic changes of ER Ca^2+^, the homeostasis of ER stress, CaMKII pathway activation, and cell apoptosis. We found that NGR1 could regulate ER Ca^2+^ via the PLC activation, providing superior neuroprotection by reducing ER stress and CaMKII pathway activation.

Our previous study showed that NGR1 (10 μmol/L) attenuates OGD/R-induced cell injury by suppressing ER stress. In this study, we sought to clarify the mechanism behind this effect. We found that OGD/R promotes the expression of p-PLCβ/p-PLCγ, and IP3R1 in neurons, and NGR1 treatment reduced the activation of p-PLCβ/p-PLCγ, and IP3R1 (Fig. 1). This finding indicates that NGR1 may inhibit the activation of PLC and IP3R1 under OGD/R. Additionally, we found that NGR1 increases the binding of GRP78 with PERK/IRE1 under OGD/R to maintain the homeostasis of ER stress, while the activator of PLC (m-3M3FBS) could block the effect of NGR1 (Fig. 2). These results show that NGR1 inhibits the activation of ER stress and that this may be achieved through PLC. PLC is an enzyme that can hydrolyze PIP2 to generate IP3, the ligand that activates IP3R^34^. As shown in a previous study, by generating IP3, PLC can promote Ca^2+^ release from the ER, leading to a high cytoplasm Ca^2+^ Concentration^35^. As a second messenger, Ca^2+^ is one of the most important regulators of ER stress; moreover, ER acts as a dynamic intracellular Ca^2+^ store and plays an important role in Ca^2+^ signaling^36,37^. It has been reported that cytoplasmic Ca^2+^ overload through IP3R can result in cytotoxicity, concomitant with the activation of ER stress^38,39^. The main subtype of Ca^2+^ channels are the IP_3_R channels, which are expressed abundantly in most cell types^40,41^. The IP3R1 is a Ca^2+^ channel on the ER and the predominant isoform in the brain among the 3 types of IP3Rs^42^.

To further explore NGR1 and its relationship with the PLC/IP3R1 pathway, cells were exposed to a PLC activator (m-3M3FBS), and a PLC inhibitor (U73122) was used for the positive control group. The concentrations of cytoplasmic Ca^2+^ and ER Ca^2+^ were detected, and the results showed that after OGD/R, there was an increased concentration of cytoplasmic Ca^2+^; NGR1 could suppress the increase of cytoplasmic Ca^2+^, but the OGD/R + NGR1 + m-3M3FBS group had a higher cytoplasmic Ca^2+^ concentration than the OGD/R + NGR1 group (Fig. 3). The endoplasmic reticulum lumen is the major Ca^2+^ store available for increasing cytosol Ca^2+^. Tg was used to trigger ER Ca^2+^ depletion, and ATP was added to trigger IP3-induced Ca^2+^ release. The results showed that NGR1 increased the ER Ca^2+^ store and inhibited the IP3-induced Ca^2+^ release under OGD/R; however, the effect of NGR1 was inhibited by m-3M3FBS. In the OGD/R + NGR1 + m-3M3FBS group, there was less Ca^2+^ stored in the ER, and ATP could easily trigger more Ca^2+^ release from the ER. Further, the results showed that m-3M3FBS treatment could independently aggravate the Ca^2+^ depletion, and U73122 could alleviate the change in Ca^2+^ concentration. Overall, these results suggest that NGR1 attenuates Ca^2+^ depletion by suppressing PLC activation (Fig. 3).

Under normal circumstances, chaperone GRP78 binds the N-termini of IRE1 and PERK, preventing their activation. When external stimuli or damage lead to ER Ca^2+^ depletion, GRP78 releases IRE1/PERK and subsequently activates ER stress^43,44^. ER stress subsequently leads to the activation of CHOP or inhibition of BCL-2 to promote cell apoptosis^45^. In our study, under OGD/R, ER stress-related proteins were activated, and the expression of BCL-2 was inhibited; however, NGR1 suppressed the activation of ER stress (Fig. 4A). With the increase of cytoplasmic Ca^2+^ (Fig. 3), CaMKII was also activated; the OGD/R group had higher expressions of p-CaMKII, p-P38, and p-JNK, but the OGD/R + NGR1 (10 μmol/L) group had lower expressions of p-CaMKII, p-P38, and p-JNK than the OGD/R group (Fig. 4B). This suggests that NGR1 also inhibits the activation of the CaMKII signal pathway. The Ca^2+^/CaMKII axis is involved in regulating cell apoptosis: Apoptosis can be triggered by increased cytoplasm Ca^2+^, and CaMKII plays a crucial role in neuron apoptosis by activating transcription factors^46^. Huang found that a CaMKII inhibitor partially prevented ischemia-induced functional deficits of cortical neurons^47^. In our study, the function of NGR1 was blocked by m-3M3FBS in the OGD/R + NGR1 + m-3M3FBS group. Examination of the proteins with Western blotting assays revealed that NGR1 suppressed OGD/R-induced activation of the ER stress and CaMKII signal pathway, but the PLC activator (m-3M3FBS) blocked the effect of NGR1. In the OGD/R + NGR1 + m-3M3FBS group, the expressions of ER stress-related proteins, p-CaMKII, p-P38, and p-JNK were higher than those in the OGD/R + NGR1 group. M-3M3FBS treatment could independently aggravate the activation of the ER stress and CaMKII pathway (Fig. 4). Further, m-3M3FBS also interfered with cell apoptosis (Fig. 5); there was more cell injury in the OGD/R + NGR1 + m-3M3FBS group than in the OGD/R + NGR1 group. All of the above data confirm that NGR1 plays a neuroprotective role via the PLC activation. NGR1 inhibits the PLC activation to attenuate the ER Ca^2+^ depletion–ER stress–CaMKII signal pathway and protects the neuron from OGD/R.

**Figure 6:**
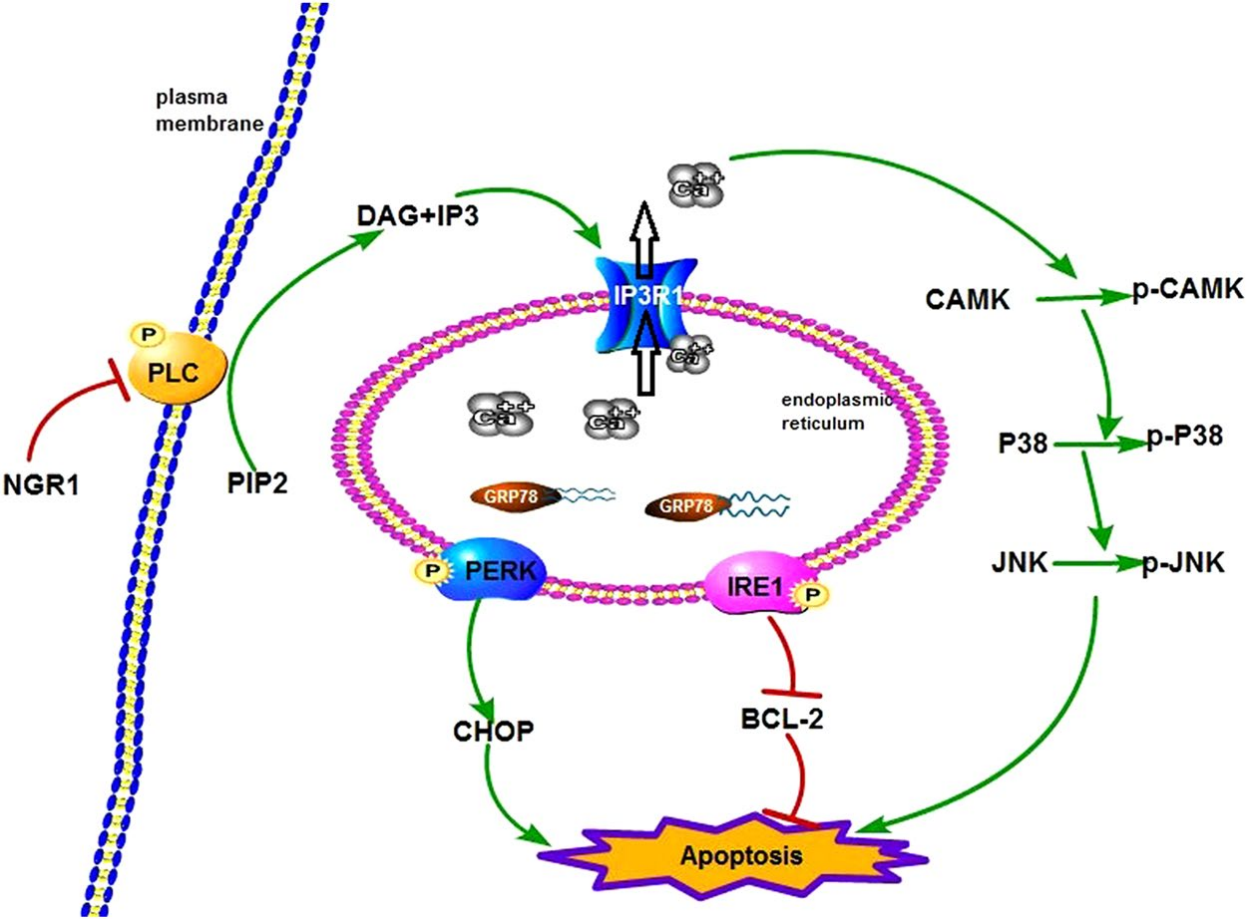
Schematic presentation of signaling mechanisms for the neuroprotective effects of NGR1. Hypoxia–ischemia results in the stimulation of PLC and PIP2 hydrolysis to IP3 and DAG, which triggers IP3-induced Ca^2+^ release and GRP78 dissociation with PERK and IRE1. This leads to the phosphorylation of PERK and IRE1. Phospho-PERK triggers the activation of CHOP, while phospho-IRE1 activates the inhibition of BCL-2. IP3-induced Ca^2+^ release also activates the phosphorylation of CaMKII, which leads to the stimulation of the p38/JNK pathway. NGR1 inhibits the stimulation of PLC and the above processes in the OGD/R case. PLC: phospholipase C; PIP2: phosphatidylinositol 4,5-bisphosphate; IP3: inositol 1,4,5-trisphosphate.

In conclusion, NGR1 can attenuate OGD/R-induced cell injury by suppressing ER stress via the PLC activation. Under hypoxia–ischemia, the PLC is activated, causing the overexpression of IP3R1, which stimulates the release of Ca^2+^ from the ER. The imbalance of Ca^2+^ in the ER leads to the breaking of bonds between PERK/IRE1 and GRP78 and subsequent activation of ER stress. Further, as a result of the increase in cytosol Ca^2+^, CaMKII is activated. NGR1 can inhibit the activation of PLC, thereby blocking the whole process and subsequent cell apoptosis (Fig. 6).

## Materials and Methods

### Drug preparation

NGR1 (chemical structure C_47_H_80_O_18_, molecular weight = 933.13, purity > 98%) was purchased from Sigma-Aldrich (St. Louis, MO, USA). m-3M3FBS (a PLC activator), thapsigargin (Tg; endoplasmic reticulum Ca^2+^-ATPase inhibitor, IP3-independent intracellular calcium releaser), and ATP were purchased from Sigma-Aldrich (St. Louis, MO, USA). U73122 (a PLC inhibitor) was purchased from Selleck (Selleck, Shanghai, China). Prior to the study, cells were administered m-3M3FBS (0, 1, 2, 5, 10, 20, 50, or 100 μmol/L) or U73122 (0, 0.1, 0.2, 0.5, 1, 2, 5, or 10 μmol/L) for MTT assay. The optimal concentrations of m-3M3FBS and U73122 were selected for further experiments. Based on the results shown in Supplemental Figure S1, we selected 20 μmol/L m-3M3FBS and 2 μmol/L U73122.

### Cell culture and drug treatment

Cortical neurons from rats were cultured as previously described^48^. Briefly, primary cortical neuron cultures were harvested from the cerebral cortices of rat fetuses (embryonic day 18; purchased from Chongqing Medical University, Chongqing, China). Cerebral cortices were removed and mechanically dissociated, then digested in 0.25% trypsin (with 0.02% EDTA) for 7 min at 37 °C. After trypsinization, the digests were centrifuged for 5 min at 1000 rpm. Cells were resuspended in Neurobasal Medium (Gibco, Gaithersburg, MD, USA) with 2% B-27 supplement (Gibco) and 2 mmol/L L-glutamine (Invitrogen, Gaithersburg, MD, USA). The cells were then subcultured in different plates, which were precoated with polyethylenimine (0.05 mg/mL, Sigma-Aldrich) for experiments, at a density of 1 × 10^4^ cells/cm^2^. Cultures were maintained in a Heraeus CO_2_ incubator (Thermo Fisher Scientific, Rockford, IL, USA) containing 5% CO_2_ and 95% air at 37 °C. Cultures were used for experiments on Day 5 *in vitro*. Cells were treated with m-3M3FBS (20 μmol/L) or U73122 (2 μmol/L) 1 h before OGD. Cells were then administered NGR1 (10 μmol/L) when exposed to oxygen–glucose deprivation and reoxygenated as previously described.

### Oxygen–glucose deprivation/reoxygenation

OGD/R was performed using primary cortical neuron cultures to mimic cerebral ischemia–reperfusion injury. Experiments were conducted on Day 5 of the cell culture. Primary cortical neurons were washed once with PBS (0.01 mol/L) and suspended in glucose-free Neurobasal Medium. Cultures were incubated at 37 °C in an anaerobic chamber (Thermo Fisher Scientific, Rockford, IL, USA) to expose them to a low-oxygen environment (1% O_2_, 5% CO_2_, and 94% N_2_). After 1.5 h, the primary cortical neurons were moved to the regular incubator with Neurobasal Medium and maintained for 24 h to mimic reperfusion.

### Calcium imaging

Cells were planted on fluorodish plates (35 mm, World Precision Instruments, Sarasota, FL, USA) for experiments. After OGD/R for 24 h, cells were washed with HBSS (calcium-free) and loaded with 5 μM Fluo-3 AM (Beyotime Institute of Biotechnology) in HBSS (calcium-free) for 60 min at 37 °C. The cells were then washed with HBSS (calcium-free) and incubated in HBSS (calcium-free) for an additional 30 min. Images were captured by an inverted confocal microscope (Live5; Carl Zeiss, Inc., Tokyo, Japan) to detect the fluorescence intensity of cells, which represented the calcium concentration in the cytoplasm. It took 90 seconds to determine basal fluorescence intensity, then 0.2 μM Tg or 2 μM of ATP were added directly to the cell solution to trigger ER Ca^2+^ depletion^49^ or IP3R- and IP3-induced Ca^2+^ release (IICR)^50^; cells were then continuously observed for 6 min. Cells were excited by a 488-nm laser, and images were acquired at 5 s intervals in time-lapse mode and subsequently analyzed using ImageJ software (National Institutes of Health, Bethesda, Maryland, USA). These data were quantified as the area under the curve (AUC) for all peaks.

### Western blotting

Protein expression was measured by Western blotting analysis. Cells were harvested by lysis buffer (Beyotime Institute of Biotechnology, Suzhou, China), and the lysate was cleared by centrifugation at 12,000 rpm for 15 min. The protein concentration was detected by a BCA protein assay kit (Beyotime Institute of Biotechnology). Equal amounts of protein samples were mixed with sodium dodecyl sulfate gel-loading buffer and heated for 5 min at 100 °C, then the protein was separated by sodium dodecyl sulfate polyacrylamide gel electrophoresis (SDS-PAGE) and transferred to a polyvinylidene fluoride (PVDF) membrane. Membranes were blocked for 90 min at room temperature in nonfat dry milk in tris-buffered saline with Tween 20 (TBST; 10 mmol/L of tris, 150 mmol/L of NaCl, pH 7.6, and 0.1% Tween 20). Membranes were incubated with a primary antibody at 4 °C overnight. Following TBST washing, all membranes were incubated with a secondary antibody for 2 h at room temperature. Bands were scanned and densitometrically analyzed by automated ImageJ software (NIH Image, Version 1.61). Band densities for the indicated phosphoproteins were normalized to the corresponding band densities for the total protein signals, and the indicated total proteins were expressed relative to β-actin signals.

### Coimmunoprecipitation assays

At 24 h after OGD/R, neurons were lysed on ice in immunoprecipitation buffer (Beyotime Institute of Biotechnology) with a protease inhibitor cocktail (Roche, Basel, Switzerland). One-fifth of the cell lysates were prepared as input samples, and the rest were used for coimmunoprecipitation. Cell lysates were pre-cleared with Protein A Sepharose beads (GE Healthcare, Uppsala, Sweden) for 1 h, and the supernatant was incubated with a primary antibody of GRP78 (Abcam, LA, USA) at 4 °C overnight. The Protein A Sepharose beads were then added to the system and incubated for 2 h at 4 °C. After incubation, the beads were washed 3 times with cold PBS. The immunoprecipitates were subjected to Western blotting analysis with an anti-PERK antibody or anti-IRE1 antibody (Abcam, LA, USA).

### Cell viability assessment

The cell survival rate tests were conducted using an MTT assay. At 24 h after OGD/R, cells were incubated with MTT (0.05 mg/L) in the Heraeus CO_2_ incubator. Four h later, the culture medium was completely removed from each well, and DMSO (100 µL) was used to dissolve the insoluble formazan crystals. The absorbance of the solvate of each well was detected by a microplate reader at 570 nm (Bio-Rad Model 680, Bio-Rad, Hercules, California, USA), and cell viability was expressed as (mean experimental absorbance/mean control absorbance) × 100%.

### Measurement of cell membrane integrity

The membrane integrity of cells was detected by the leakage of LDH. The supernatants of all of the wells were collected, and the cells of the respective well were lysed by 0.5% Triton X-100 for 5 min. The LDH content of the supernatant and cell lysis solution were measured by the LDH assay kit in accordance with the manufacturer’s instructions (Nanjing Jiancheng Biological Engineering, Nanjing, China). The level of LDH release was expressed as (supernatant LDH activity/whole LDH activity) × 100%.

### Terminal deoxynucleoitidyl transferase-mediated nick-end labeling (TUNEL) and nuclear staining

Cortical neurons were placed in plates that had positioned slides. After administration, the cell culture medium was removed, and the rest of cells were washed with PBS (0.01 M, pH 7.4). Cells were then prepared with 4% paraformaldehyde (pH 7.4) for 1 h, 3% H_2_O_2_ in methanol for 10 min, and 0.2% Triton X-100 for 3 min on ice. After PBS washing, a TUNEL reaction mixture (Thermo Fisher Scientific, Rockford, IL, USA) was added, and samples were incubated for 60 min at 37 °C in a humidified environment in the dark. This was followed by incubation in 4,5-diamino-2-phenylindole (DAPI) for 2 min. Samples were examined under a fluorescence microscope (Olympus, Tokyo, Japan) using an excitation wavelength of 450–500 nm (green) and a detection of excitation wavelength of 320–400 nm (blue). The proportion of TUNEL-positive cell nuclei (green) to the number of total nuclei (blue) was then calculated.

### Statistical Analysis

All data are expressed as mean ± standard deviation (*SD*). Statistical analyses were performed using SPSS version 17.0 (SPSS, Chicago, IL, USA). Analyses between different groups were performed using the two-tailed Student’s *t* test or one-way analysis of variance. A *p* value below 0.05 was regarded as statistically significant.

## Electronic supplementary material

supplementary information
