# Supplementary material for: Notoginsenoside R1 Alleviates Oxygen–Glucose Deprivation/Reoxygenation Injury by Suppressing Endoplasmic Reticulum Calcium Release via PLC
Source: Sci Rep. 2017 Nov 24;7:16226. doi: 10.1038/s41598-017-16373-7 (PMC5701215; doi:10.1038/s41598-017-16373-7)
Supplement: Supplementary file 1 — supplementary information [file 41598_2017_16373_MOESM1_ESM.pdf]

Notoginsenoside R1 Alleviates Oxygen–Glucose Deprivation/Reoxygenation Injury by Suppressing Endoplasmic Reticulum Calcium Release via PLC

Yan Wang[1], Liu Tu[1], Yingbo Li[1], Di Chen[1], ZhaoLiu[2], Xuelian Hu[3], Shali Wang[1*]

[1]Cerebrovascular Diseases Laboratory, Institute of Neuroscience, Chongqing Medical University, Chongqing,400016, China

[2]Department of Neurology, Chongqing General Hospital, Chongqing, 400013, China

[3]Department of Pharmacy,Second Affiliated Hospital, Third Military Medical University, Chongqing, 400037, China

[*]Correspondence author:

Cerebrovascular Diseases Laboratory, Institute of Neuroscience, Chongqing Medical University, 1 Yixueyuan Road, Yuzhong District, Chongqing 400016, China (S. Wang);

Tel: +86-023-68068920, Fax: +86-023-68892728

E-mail addresses: 63557408@qq.com； cqykdxwangshali@cqmu.edu.cn

Supplementary Figure S1

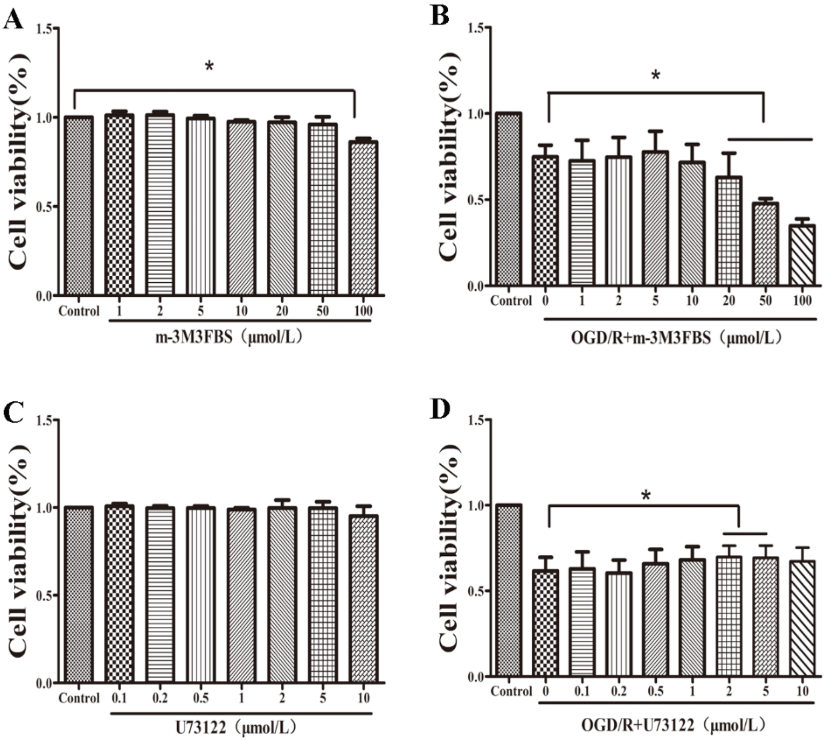

Suppl Fig.S1. Concentration-dependent effects of m-3M3FBS or U73122 on cell viability at normal or after OGD/R

Supplementary Figure S2

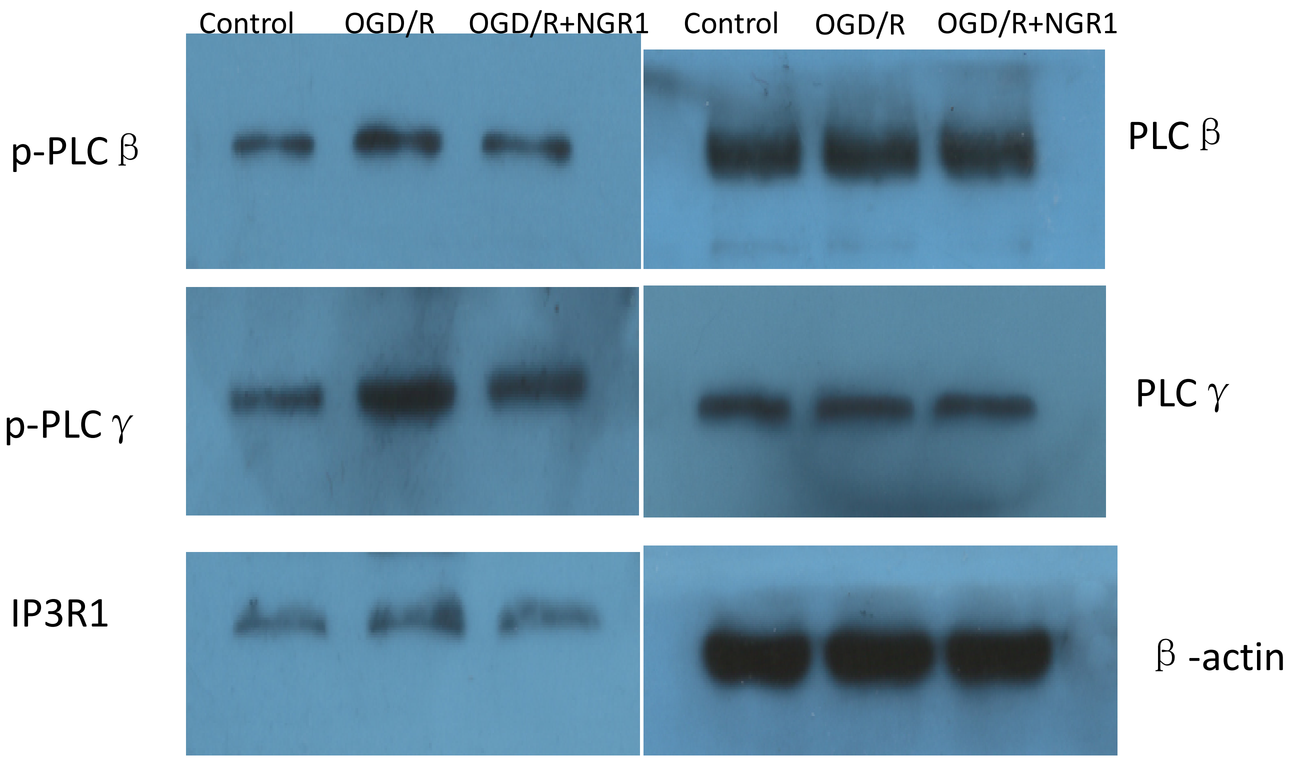

Suppl Fig.S2. Original picture of the cropped blots shown in Figure 1.

Supplementary Figure S3

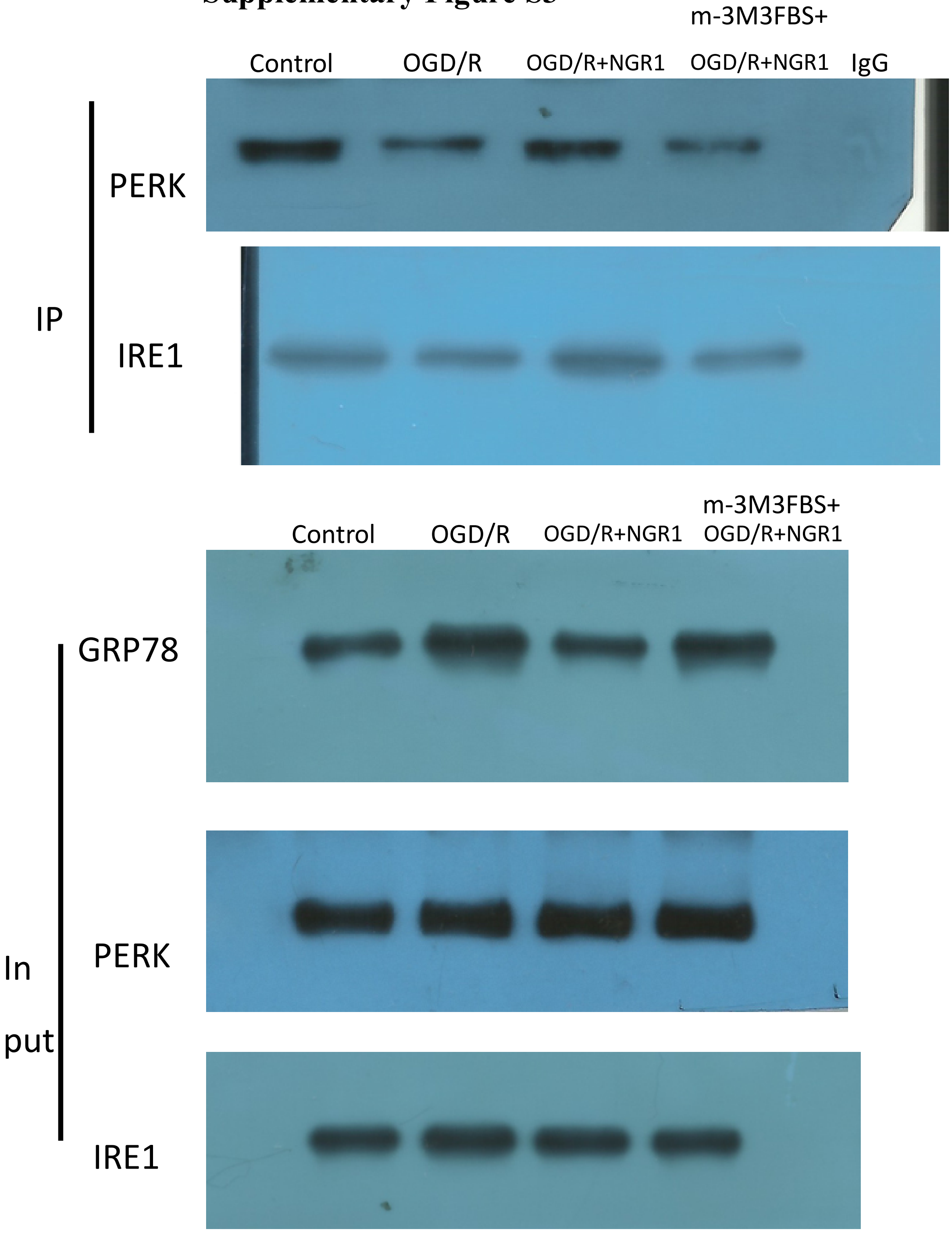

Suppl Fig.S3. Original picture of the cropped blots shown in Figure 2.

Supplementary Figure S4-A

1: Control 2:OGD/R 3: OGD/R+NGR1 4: OGD/R+NGR1+ m-3M3FBS

5: OGD/R+ m-3M3FBS 6：OGD/R+U73122

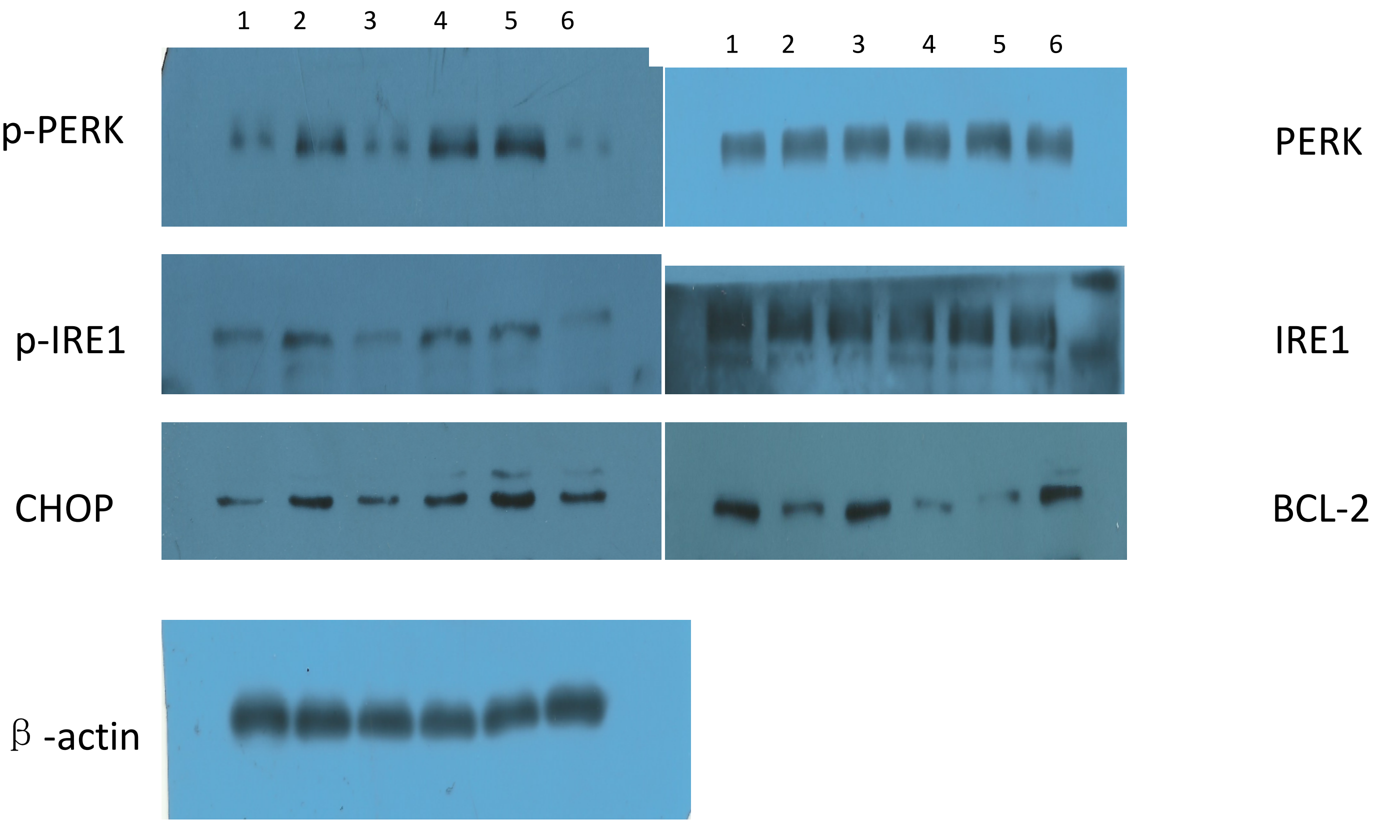

Supplementary Figure S4-B

1: Control 2:OGD/R 3: OGD/R+NGR1 4: OGD/R+NGR1+ m-3M3FBS

5: OGD/R+ m-3M3FBS 6：OGD/R+U73122

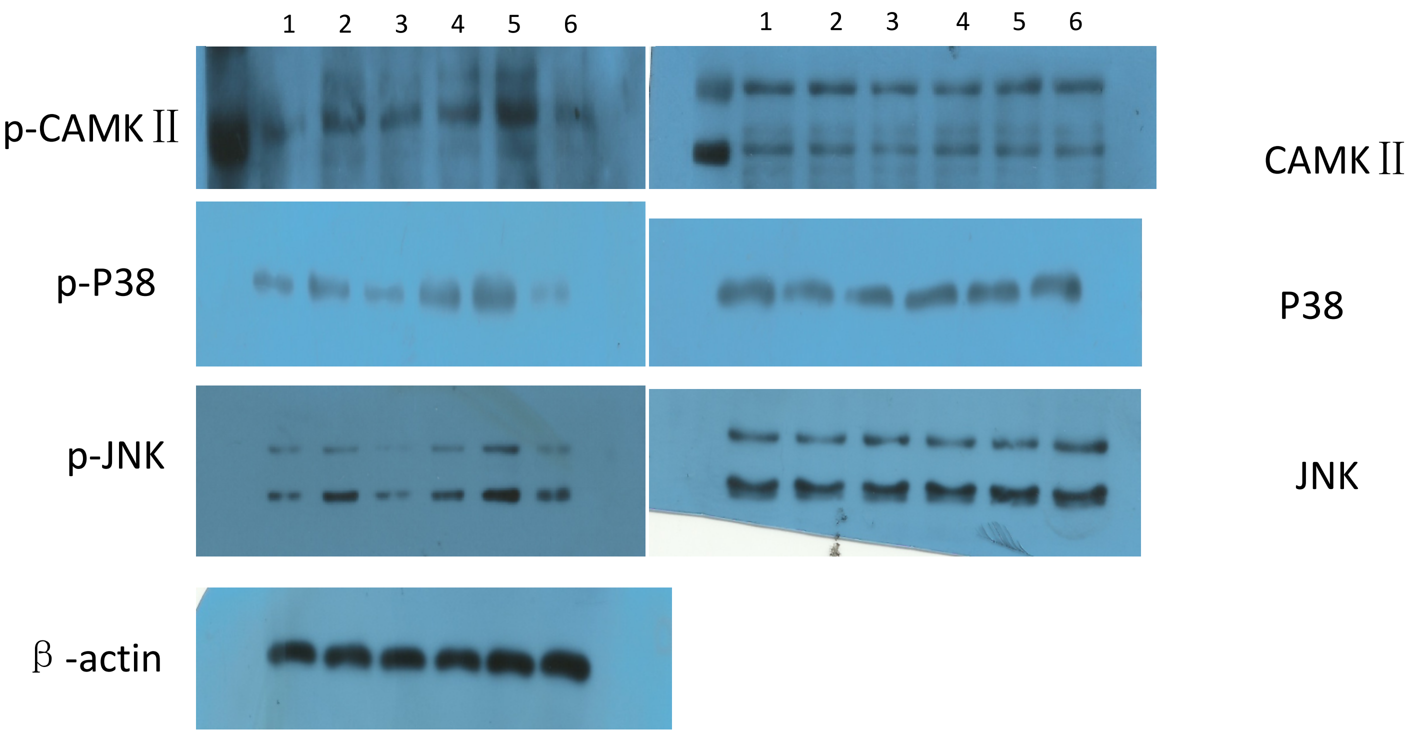

Suppl Fig.S4. Original picture of the cropped blots shown in Figure 4.
